# Some Facts Relative to the Spheroidal State of Bodies — Trial by Fire — Man Incombustible

**Published:** 1850-01

**Authors:** 


### ECLECTIC DEPARTMENT,

### AND SPIRIT OF THE MEDICAL PERIODICAL PRESS.

---

*Some facts relative to the Spheroidal state of Bodies—Trial by Fire—Man Incombustible.* By P. N. Boutigny, Reposted to Academie de Sciences. (Southern Literary Messenger.)

"Upon my return home," says M. Boutigny, "I did not fail to inquire of the workmen what would happen if the finger were immersed in the incandesent mass of melted iron? Most of them laughed in my face. But that did not discourage me. After a while, being at the forge of Magny, near Lure, I repeated my question to a workman, who replied that "nothing was more simple;" and to prove it, he instantly passed his fingers into the incandescent column of ore which was just then issuing from a Wilkinson. Another workman who stood by, performed the same experiment with equal impunity. Emboldened by what I saw, I did the same."

The fact in question was no longer doubtful, but M. Boutigny hesitated to communicate it to the Academy until he should be prepared to support it by the adduction of various other experiments. These experiments he thus describes: "I cut or divided with one hand a spout of melted ore five or six centimeters (abont two inches) in diameter, as it issued from the furnace; and plunged the other into a vessel filled with the incandescent liquid, which it was really frightful to behold. I shuddered involuntarily. But both hands issued victorious from the trial; and now, if any thing appears surprising to me, it is that similar experiments are not of every day occurrence. Certainly it will be asked what precaution should be taken to guaranty the hand from the action of the burning fluid? I answer none! Fear not. Perform the experiment with confidence. Pass the hand rapidly, yet not too rapidly, into the molten mass. If the experiment is made timidly, and with too great rapidity, you may overcome the repulsive force which exists in incandescent bodies, and thus sstablish contact with the skin. In that case the skin would indubitably remain there, and in a condition not difficult to conceive. The experiment succeeds particularly well when the skin is moist. The involuntary terror which one experiences in presence of these masses of fire, almost always puts the whole body in that condition of moisture essential to success. The following I have found to be the best preparation for the experiment. I rub my hands with soap, so as to give them a polished surface. Then at the moment of making the experiment, I plunge the hand into a cold solution of sal ammoniac saturated with sulphurous acid, or simply into water containing sal ammoniac, or if you have not the latter substance convenient, dip the hand merely in cold water."

M. Boutigny then gives the following philosophic explanation of this phenomenon:

"It is to my mind a positively established fact that the hand and metal do not come in contact with each other. If there be no contact, heating can only take place by means of radiation. This is enormous, it must be admitted; but in our experiment no account need be taken of radiation, for, in fact, it is nullified by reflection. I think that I have long since proved that water in the spheroidal state possesses the remarkable property of reflecting the calorific rays, and that its temperature never reaches that of its boiling point; whence it follows that the finger or the hand, being moist, cannot attain the temperature of 100°, the experiment not being of sufficiently long duration to permit the complete evaporation of its moisture to be effected. Persons familiar with the experiment of immersing in water a body of incandescent silver or platina, will readily understand the mechanism of this. In the first case it is the water retiring from the metal which then seems to be enclosed within a crystal envelope; in the second case it is the liquid metal which retires from the moist hand. In the first place the metal is active and the water passive; in the second, the moistened hand is active, and the fused metal is passive. It is the same experiment reversed; and the two form but one. In one word, the hand, inserted in metal in a state of fusion, isolates itself. The humidity which covers it passing to the spheroidal state, reflects the radiant caloric, and is not heated sufficiently to boil. It is true, therefore, as I said in the beginning, that this experiment, apparently so dangerous, is in fact, almost absolutely without danger. I have often repeated it with lead, bronze, &c., and invariably with the same success. Thus in the course of ten years I have made ice in a furnace heated to whiteness, and have bathed with impunity in a mass of incandescent metal; and that by virtue of the laws which govern matter in the spheroidal state. It results also from these notes, that a considerable number of facts reported in history, and generally deemed fabulous, may well be true. Ancient philosophers probably knew much that we are now ignorant of. A little more respect for them, and a little less admiration for ourselves would do us no harm."

The pretended miracle by which one of the Eastern Magi, disciples of Zoroaster, is narrated to have gained thousands of converts, is now of easy solution. He proposed that twenty pounds of molten brass should be poured hot from the furnace upon his naked body, upon condition that if he underwent the trial uninjured, unbelievers, constrained by the prodigy, would profess conversion to the faith. It was done, and the scientific imposter witnessed the rapid acceptance of his creed.

[We shall be satisfied to leave these experiments to others.]—Ed.

